# Relationship of increased aurora kinase A gene copy number, prognosis and response to chemotherapy in patients with metastatic colorectal cancer

**DOI:** 10.1038/bjc.2011.587

**Published:** 2012-01-12

**Authors:** E Dotan, N J Meropol, F Zhu, F Zambito, B Bove, K Q Cai, A K Godwin, E A Golemis, I Astsaturov, S J Cohen

**Affiliations:** 1 Department of Medical Oncology, Fox Chase Cancer Center, 333 Cottman Avenue, Philadelphia, PA 19111, USA; 2 University Hospitals Seidman Cancer Center, Case Comprehensive Cancer Center, Case Western Reserve University, 11100 Euclid Avenue, Cleveland, OH 44106, USA; 3 Department of Biostatistics and Bioinformatics, Fox Chase Cancer Center, 333 Cottman Avenue, Philadelphia, PA 1911, USA; 4 Clinical Molecular Genetics Laboratory Facility, Fox Chase Cancer Center, 333 Cottman Avenue, Philadelphia, PA 19111, USA; 5 Biosample Repository Core Facility, Fox Chase Cancer Center, 333 Cottman Avenue, Philadelphia, PA 19111, USA; 6 Department of Pathology and Laboratory Medicine, University of Kansas Medical Center, 3901 Rainbow Boulevard, Kansas City, KS 66160, USA; 7 Program in Developmental Therapeutics, Fox Chase Cancer Center, 333 Cottman Avenue, Philadelphia, PA 19111, USA

**Keywords:** colon cancer, aurora kinase A, copy number, *KRAS* mutation

## Abstract

**Background:**

Increased Aurora kinase A gene copy number (*AURKA-CN*) has been reported in metastatic colorectal cancer (mCRC), with unknown relationship to clinical outcome. We correlated increased *AURKA-CN* in mCRC tumours with *KRAS* mutation status, overall and progression-free survival (OS, PFS).

**Methods:**

Sixty-one mCRC tumours were analysed for *AURKA-CN* using q-PCR, and *KRAS* mutation status by direct sequencing. Expression of AURKA protein was analysed by immunohistochemistry. Cox-proportional hazard method, Kaplan–Meier curves and log-rank statistics were used to estimate and compare the hazard ratios and median survival between the groups.

**Results:**

In all, 68% of tumour exhibited high *AURKA-CN*, and 29% had a *KRAS* mutation, without correlation between the two. Patients with high AURKA-CN tumours had longer median OS (48.6 *vs* 18.8 months, *P*=0.01), with stronger trend among *KRAS* wild-type tumours (median OS not reached *vs* 18.8 months, *P*=0.003). Progression-free survival was longer on first-line or second-line chemotherapy among patients with *KRAS* wild-type and high *vs* low *AURKA-CN* (first: 17.6 *vs* 5.13 months, *P*=0.04; second: 10.4 *vs* 5.1 months, *P*=0.01). *AURKA-CN* level did not affect outcomes among patients with *KRAS* mutant tumours.

**Conclusion:**

Increased *AURKA-CN* is common in mCRC tumours and is associated with longer OS and longer PFS during chemotherapy, particularly in *KRAS* wild-type tumours.

Aurora kinases (Aurora A, B and C) are important regulatory proteins of the mitotic process (Goepfert and Brinkley, 2000; Sen *et al*, 2008). Due to their crucial function in cell division, these proteins have been extensively studied in many cancers for their role in carcinogenesis and as potential treatment targets (Zeng *et al*, 2007; Nadler *et al*, 2008; Park *et al*, 2008; Qu *et al*, 2008; Tanaka *et al*, 2008; Wan *et al*, 2008). Aurora kinase A (AURKA) (also known as Aurora-2, BTAK/STK15) regulates mitotic entry, centrosome maturation, bipolar spindle assembly, chromosome alignment, cytokinesis and exit from mitosis (Berdnik and Knoblich, 2002; Hirota *et al*, 2003; Marumoto *et al*, 2003; Dutertre *et al*, 2004). Aberrations in the function of Aurora kinases can result in abnormal cell division and aneuploidy due to losses or gains of whole chromosomes (Marumoto *et al*, 2003). Amplification of AURKA has been shown to induce the formation of a multipolar mitotic spindle, which results in abnormal chromosome alignment and cell division (Meraldi *et al*, 2002). Resultant genomic instability, aneuploidy and hyperploidy can promote tumour development.

In colorectal cancer (CRC), AURKA protein overexpression and amplification have been frequently observed. Bischoff *et al* (1998) were the first to report overexpression of AURKA mRNA in >50% of CRC tumours. Baba *et al* (2009) later reported overexpression of AURKA protein by immunohistochemistry (IHC) in 19% of CRC samples of various disease stages. In a multivariate analysis, AURKA protein overexpression was associated with chromosomal instability (identified as loss of heterozygosity in 2p, 5q, 17q and 18q) but did not correlate with clinical outcomes. Lam *et al* (2008) identified AURKA overexpression by IHC in 48.5% of early-stage CRC samples. This finding was associated with well/moderately differentiated tumours (*P*=0.04), tumours of the distal colon (*P*=0.01) and non-mucinous histology (*P*=0.001). However, no association with clinical outcomes was detected.

The group led by Nishida *et al* (2007) was the first to demonstrate an increase in *AURKA* gene copy number (*AURKA-CN*) in 29% of a small sample of colorectal tumours. This study demonstrated a strong correlation between increased *AURKA-CN* and chromosomal instability, and no association between increased *AURKA-CN* and *KRAS* mutation status. Recently, the group led by Zhang *et al* (2010) reported increased *AURKA-CN* in 32% of CRC tumour samples and particularly in higher stage tumours, suggesting that *AURKA* may have a role in tumour progression. To date, the finding of increased *AURKA-CN* has not been correlated with clinical outcomes of patients with CRC.

In recent years, the *KRAS* pathway has been proven to have an important predictive role in the treatment of CRC (Cunningham *et al*, 2004; Van Cutsem *et al*, 2007; Karapetis *et al*, 2008; Douillard *et al*, 2010; Bokemeyer *et al*, 2011). Anti-EGFR therapy is effective and approved for the management of metastatic CRC (mCRC) without *KRAS* mutations. Earlier work by our group has demonstrated synergistic lethality achieved by inhibition of both the Aurora and the *KRAS* pathways on CRC tissue samples (Astsaturov *et al*, 2010). The goal in this study was to assess the frequency of increased *AURKA-CN* in archival tumour tissues of patients with metastatic CRC (mCRC) and correlate this finding with progression-free survival (PFS), overall survival (OS), and *KRAS* mutation status.

## Patients and Methods

### Patient samples

We analysed 61 consecutive mCRC tumour samples submitted between 2006 and 2009 to the Fox Chase Cancer Center (FCCC) molecular genetic facility for evaluation of *KRAS* mutation status, with remaining tissue available for *AURKA-CN* evaluation. Most of the patients had their tumour samples submitted by their treating oncologist for *KRAS* mutation status evaluation after the test became routinely used in clinical practice (2008). Twenty patients in this cohort were enrolled on an institutional phase II study evaluating the combination of capecitabine, oxaliplatin, cetuximab and bevacizumab in the front line metastatic setting (between 2006 and 2008). These patients submitted tissue samples for future research at time of study enrollment, which was used for this analysis. Overall survival data were available for 59 out of 61 patients. Fifty-three patients had received chemotherapy treatment at FCCC and were evaluable for PFS. Progression-free survival was defined as the time that elapsed from treatment initiation until evidence of progressive disease or death. For the purpose of this analysis, deintensification of therapy for adverse events, or planned treatment breaks were considered as same line of therapy.

### Evaluation of *AURKA-CN*

We utilised a quantitative genomic PCR method described by Nishida *et al* (2007) to evaluate the *AURKA* gene copy number. In all, 20 ng of genomic DNA purified from paraffin-embedded tissue (PET) sections using the QIAamp DNA Mini Kit (QIAGEN, Valencia, CA, USA) was used. A real-time PCR method was used to determine copy number alterations in the *AURKA* gene at the Fox Chase Genomics and Genetics Facility using an ABI Prism 7900 sequence detection system (Applied Biosystems, Foster City, CA, USA). The primer and probe sequences for genomic real-time PCR for each of the genes were as follows: the Aurora A forward primer: 5′-TCTTTTATAGAAATGTGTGGAAGTTCCT-3′ reverse primer: 5′-CAATAAAAAAGTACAGACGCATAAACCA-3′ probe: 5′-CTGTCCTTAGAAATAACCACTAC-3′. All probes were labelled with FAM as the reporter dye and TAMRA as the quencher. Each PCR amplification reaction was performed in triplicate to ensure accurate results. The RNAse-P used was the endogenous reference gene, for which a set primer probe was purchased from Applied Biosystems. Genomic DNA isolated from DLD1 or HCT116 cell lines served as negative controls for *AURKA* gene amplification. DNA from the Caco-2 cell line provided a positive control as the *AURKA* gene locus in this cell line has been reported as four-fold amplified (Nishida *et al*, 2007).

### *KRAS* mutation status evaluation

Tumour samples were tested for the presence of a *KRAS* mutation on codons 12 and 13. DNA analyses were performed within the Fox Chase Clinical Molecular Genetics Laboratory. Extraction, isolation and purification of DNA were performed from formalin-fixed PET suitable for molecular analysis using the WaxFree DNA extraction kit (WF-100; TrimGen, Sparks, MD, USA). Ten to fifteen fresh cut unstained slides were used for analysis. DNA (∼10 ng) was amplified by PCR using primer sequences located on either side of the region of the coding exon of interest. PCR products were detected by agarose gel electrophoresis. Mutations were detected by sequencing of the purified PCR amplified product (BigDye Terminator v.1.1 Cycle Sequencing Kit; Applied Biosystems) and evaluated by capillary electrophoresis (ABI 3100; Applied Biosystems).

### IHC evaluation of AURKA phosphorylation

Activation of AURKA occurs through autophosphorylation of a threonine-288 residue. We evaluated the hyperactive state of the AURKA by IHC with antibodies specific to the autophosphorylated threonine-288 residue (Aurora A phospho-T288, Abcam (Cambridge, MA, USA), rabbit primer, Cat. AB58494) (Walter *et al*, 2000). Immunohistochemical staining was performed on 5 μm sections. After deparaffinisation and rehydration, sections were subjected to heat-induced epitope retrieval by immersion in a 0.01 M citrate buffer (pH 6.0). Endogenous peroxidase activity was blocked for 15 min in 3% hydrogen peroxide in methanol. Non-specific binding was blocked by treatment with a blocking reagent (Protein Block Serum-Free; Dako, Carpinteria, CA, USA) for 30 min at room temperature. Immunodetection was performed with the Dako Envision+ system. The antigen–antibody immunoreaction was visualised using 3-3′-diaminobenzidine as the chromogen. The slides were washed, counterstained with haematoxylin, dehydrated with alcohol, cleared in xylene and mounted. Patient samples that were shown previously to express high levels of phospho-T288 Aurora A (pAurora A) were used as positive controls. The negative control was performed by replacing the primary antibody with negative control rabbit IgG.

Evaluation of the staining intensity and positive cell numbers of pAurora A was performed by a pathologist (KC). Tumours with an intermediate or strong nuclear pAurora A immunoreaction in >10% of the tumour cells were scored as pAurora positive. Lack of staining or staining <10% of tumour cells was scored as negative (Lam *et al*, 2008).

### Clinical data collection

A clinicopathological database was created and included demographic information, stage at presentation, treatment regimens, response to therapies and survival. Clinical data were obtained through medical record review using the electronic medical record at FCCC. All patient data were coded and all identifiers were removed before analysis. The study was approved by FCCC's Institutional Review Board.

### Statistical analysis

Sixty-one tissue samples of mCRC patients at FCCC were included in this analysis. The distribution of *AURKA-CN* was examined. Full survival information was available for 59 patients. The distribution of *AURKA-CN* was examined. There are outliers with very high values. To avoid the large influence from the outliers while studying the association between *AURKA-CN* and survival, we delineated the values by quartile. Kaplan–Meier curves were constructed and log-rank test analysis showed a significant association (results not shown). After confirming this association, we conducted an analysis to search for the most sensitive cutoff for the definition of ‘high’ *AURKA-CN* based on our samples. From the Kaplan–Meier curves stratified by quartiles, we found that only the group with *AURKA-CN* in the first quartile showed a large separation from the remainder of the cohort. The distribution of *AURKA-CN* was then summarised based on the percentile of the values and used to create data sets with various cutoff points. We examined the data sets with cutoffs from the 25th percentile to 50th percentile. For each data set, Kaplan–Meier curves were created and compared using log-rank statistics and Cox-proportional hazard model. Considering our outcome was longitudinal and traditional area under the ROC curve analyses do not taken into account the varied follow-up time, we utilised the C index (Harrell *et al*, 1982, 1996) which is a member of the ‘Kendall family’ of rank parameters and is constantly used to estimate the concordance probability with censored data. Like AUC, a value of 0.5 implies complete discordance. Higher values suggest higher concordance between the data and the predicted values from the model. This statistical analysis enabled us to determine the best cutoff points by comprehensively considering hazard ratios (HRs), *P*-values, separation between Kaplan-Meier curves and C index. After we determined the best cutoff points, we compared the baseline demographics and treatment using a *t*-test or *χ*^2^-test depending on whether the variable under consideration was continuous or categorical. As the groups were well balanced, no further adjustment for confounding factors was performed.

## Results

### Patient and treatment characteristics

Table 1 summarises the characteristics and treatment of patients whose tumours were included in this cohort. The majority of patients in this analysis had initially presented with metastatic disease and were treated with FOLFOX (infusional and bolus 5-fluoruracil, leucovorin and oxaliplatin) plus bevacizumab. All characteristics were well balanced between high and low *AURKA-CN* cohorts (Table 1). The use of various chemotherapeutic agents was also well balanced between the groups with the exception of bevacizumab, which was used more commonly in the low *AURKA-CN* group in the second-line setting.

### Determination and frequency of high *AURKA-CN* and *KRAS*

**Figure 1:**
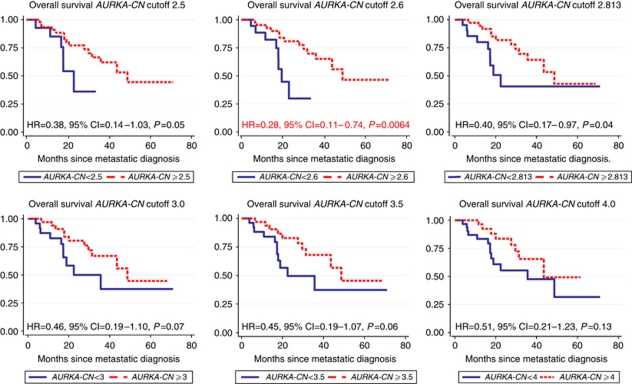
Kaplan–Meier survival estimates by *AURKA-CN* and various cutoff values.

Of the 61 tissue samples obtained for *AURKA-CN* analysis, 62% originated from primary tumours, while 38% originated from metastatic sites. This distribution was similar between the high *AURKA-CN* group and the low *AURKA-CN* group (*P*=0.925). In the analysis of *AURKA-CN*, we viewed any value >2 as representing abnormal expression of the *AURKA* gene. We thus created multiple data sets using values selected from percentile tables. These data sets were each analysed using various cutoff points to determine the one most predictive of clinical outcomes. An OS analysis demonstrated statistically significant differences in OS using multiple cutoff values <3.0 (Table 2; Figure 1). The percentile analysis demonstrated a cutoff of 2.6 (defining approximately the 30th percentile; Table 2) as the most sensitive and this was selected as the cutoff value used to define high *vs* low *AURKA-CN* for further analyses.

**Figure 2:**
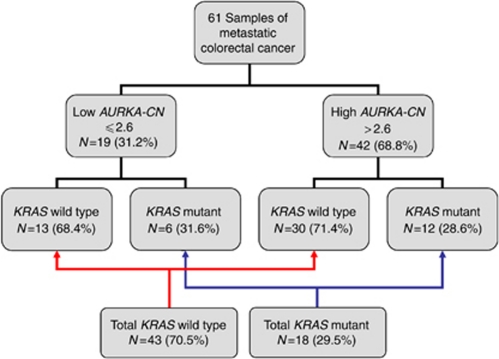
Frequency of *KRAS* mutations by *AURKA-CN.*

By these criteria, 42 of 61 (68.85%) samples expressed increased *AURKA-CN*. *AURKA-CN* values ranged from 0.122 to 23.73 (mean 4.875, s.d. 3.96). A *KRAS* mutation was identified in 18 of 61 samples (29.5%). The most common mutation noted was in codon 12 (16 samples, 89%) with two patient tumours having a mutation in codon 13. No correlation was noted between *KRAS* mutation status and the presence of high or low *AURKA-CN* (*P*=0.81; Figure 2).

### *AURKA-CN, KRAS* and PFS

**Figure 3:**
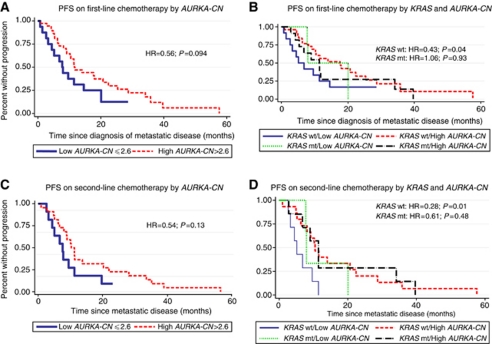
Progression-free survival (PFS) of metastatic colorectal cancer patients receiving first-line and second-line chemotherapy by *AURKA-CN* and *KRAS* mutation status. (**A**) PFS on first-line chemotherapy by *AURKA-CN*; (**B**) PFS on first-line chemotherapy by *AURKA-CN* and *KRAS* mutation status; (**C**) PFS on second-line chemotherapy by *AURKA-CN*; and (**D**) PFS on second-line chemotherapy by *AURKA-CN* and *KRAS* mutation status.

Complete treatment information was available for 53 out of 61 patients, while eight patients were lost to follow-up or elected to receive treatment at an outside institution. The median PFS on first-line chemotherapy was 11.4 months for the full cohort. For patients with high *AURKA-CN* tumours, the median PFS on first-line chemotherapy was 11.5 months *vs* 7.7 months for patients with low *AURKA-CN* tumours (HR=0.56, 95% CI: 0.28–1.1, *P*=0.094; Figure 3A). Among patients with *KRAS* wild-type tumours, those with high *AURKA-CN* tumours had prolonged PFS compared with patients with low *AURKA-CN* tumours (HR=0.43, 95% CI: 0.19–0.94, *P*=0.04; Figure 3B). Conversely, PFS did not differ by *AURKA-CN* expression among patients with *KRAS* mutant tumours (HR=1.06, 95% CI: 0.28–3.93, *P*=0.93), although the small sample size limits definitive conclusions regarding this population.

Thirty-three patients in our cohort who received second-line chemotherapy were evaluable for PFS. The median PFS on second-line chemotherapy was 9.1 months for the full cohort. No statistically significant difference in PFS was noted among patients with high *vs* low *AURKA-CN* tumours in the group overall (10.4 months *vs* 7.7 months; HR=0.54, 95% CI: 0.24–1.19, *P*=0.13; Figure 3C). However, as with response to first-line treatment, patients with high *AURKA-CN* and *KRAS* wild-type status had an improved PFS compared with patients with low *AURKA-CN* and *KRAS* wild-type status (10.4 months *vs* 5.1 months; HR=0.28, 95% CI: 0.11–0.74, *P*=0.01; Figure 3D).

We next performed an exploratory analysis to determine if the relationship of *AURKA-CN* to PFS was more pronounced in patients receiving the anti-EGFR antibody cetuximab. Table 3 outlines median PFS and HRs for high and low *AURKA-CN* tumours by *KRAS* mutational status and the use of cetuximab. Interestingly, among patients with *KRAS* wild-type tumours, high *AURKA-CN* appeared to have the largest association with outcome among patients who did not receive cetuximab. However, small numbers preclude any definitive conclusion.

### *AURKA-CN, KRAS* and OS

**Figure 4:**
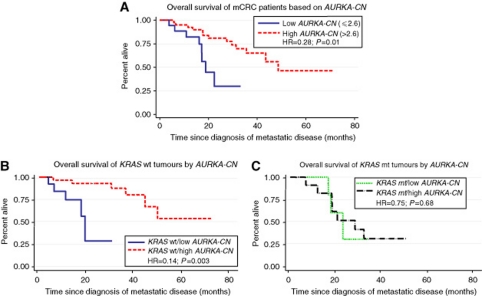
Overall survival of metastatic colorectal cancer patients by *AURKA-CN* for entire cohort (**A**), the *KRAS* wild-type population (**B**) and the *KRAS* mutant population (**C**).

The median follow-up of patients in this cohort was 742 days (range 173–3229 days). When using quartiles, one quartile increase of *AURKA-CN* reduced the hazard by 0.63 (95% CI: 0.40–0.97, *P*=0.04). The C index is the highest at a cutoff of 2.813. However, the *P*-values for Cox model and log-rank tests were barely significant. The C index for cutoff at 2.6 is very similar while the HR is much lower. Thus, we decided to use a cutoff of 2.6 for the rest of the analysis. A significantly longer OS was noted among patients with high *vs* low *AURKA-CN* (median OS 48.6 months for patients with high *AURKA-CN* tumours compared with 18.8 months for patients with low *AURKA-CN* tumours, HR=0.28, 95% CI: 0.10–0.73, *P*=0.01; Figure 4A). In all, 1-year and 2-year survival were also longer among patients with high *AURKA-CN* tumours compared with those with low *AURKA-CN* tumours (1 year: 92.5% *vs* 82.2% *P*<0.001; 2 year: 80.9% *vs* 29.9%, *P*<0.001).

Similarly to PFS, the longer OS for patients with high *AURKA-CN* tumours was particularly pronounced in the *KRAS* wild-type patient population (Figure 4B). Among patients with *KRAS* wild-type tumours, the median OS was not reached for high *AURKA-CN* compared with 18.8 months for the group with low *AURKA-CN* tumours (HR=0.14, 95% CI: 0.038–0.514, *P*=0.003). Similarly, increased 1-year and 2-year survival was seen among those patients with *KRAS* wild-type tumours and high *AURKA-CN* compared with patients with *KRAS* wild-type tumours and low *AURKA-CN* (1 year: 96% *vs* 75% *P*<0.0001; 2 year: 93% *vs* 28% *P*<0.0001). No difference in survival was noted by *AURKA-CN* status in the *KRAS* mutated population (Figure 4C, HR=0.75, 95% CI: 0.18–2.98, *P*=0.68).

### IHC evaluation of AURKA hyperactivity

**Figure 5:**
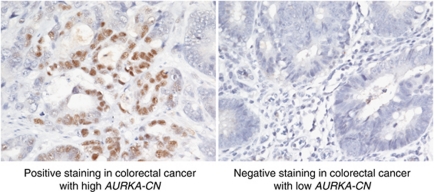
Immunohistochemistry staining for phosphorylated AURKA in metastatic colorectal cancer samples. Activated state of the Aurora A kinase was evaluated using specific antibodies to the phosphorylated threonine-288 residue (Aurora A phospho-T288). Positive staining (>10% nuclear staining) is displayed on the left and negative staining is displayed on the right.

Immunohistochemical evaluation of phosphorylated AURKA protein was performed on 27 available tumour samples (20 with high *AURKA-CN* and 7 with low *AURKA-CN*). Among the seven tumours with low *AURKA-CN*, none were positive for phosphorylated T-288. However, among the 20 tumours with high *AURKA-CN*, 9 were found to have positive staining (*P*=0.06 for difference between high and low *AURKA-CN* tumours). A representative image of positive and negative phosphorylated AURKA staining is provided in Figure 5.

## Discussion

Although five classes of systemic agents are available for the treatment of advanced CRC, the majority of patients are not cured and median survival has plateaued near 2 years (Saltz *et al*, 2008; Hecht *et al*, 2009; Tol *et al*, 2009; Jemal *et al*, 2010; American Cancer Society, 2010). Thus, the search for novel targets such as AURKA is of critical importance. Better understanding of the role of the Aurora kinases and their activity in CRC would support the development of new treatment modalities targeting this pathway. Previous groups have studied the frequency of increased *AURKA-CN* in CRC samples using various cutoff values for defining high *AURKA-CN*. We performed a novel statistical analysis incorporating the HRs, *P*-values and Kaplan–Meier survival curves to determine the most sensitive cutoff value. Our analysis demonstrated the most sensitive cutoff value to be 2.6, which resulted in identification of 68% of mCRC samples as having high *AURKA-CN*. The groups led by Nishida *et al* (2007) and Zhang *et al* (2010) reported increased *AURKA-CN* in ∼30% of CRC tumours. The differences in frequency may simply reflect alternate methodology and cutoffs. The two aforementioned studies were conducted in an Asian patient population with all stages of disease. Differences in patient population and stage of disease studied may thus also account for differences in *AURKA-CN* frequency.

The role of Aurora kinase in tumourigenesis is well documented, although its utility as a prognostic marker is still under investigation in many cancers. Nadler *et al* (2008) reported a high expression of Aurora A by AQUA to be a poor prognostic marker in patients with breast cancer. Similarly overexpression of Aurora kinase B was found to be a poor prognostic marker in non-small cell lung cancer (Smith *et al*, 2005). In contrast, our report in mCRC is the first demonstrating an association between high *AURKA-CN* and longer OS. A few groups have previously been unable to demonstrate a correlation between AURKA protein expression by IHC and clinical outcome. Similarly to our findings, Lam and colleagues have demonstrated higher detection of the AURKA protein by IHC in low-grade (well or moderately differentiated) CRC samples compared with high-grade (poorly differentiated) tumours. Therefore, high *AURKA-CN* may be a marker of a less aggressive biology with improved clinical outcome. Alternatively, increased *AURKA-CN* may predict for improved benefit from chemotherapy by rendering the cells more susceptible, due to abnormal cell divisions. Our data demonstrating increased PFS for patients with high *AURKA-CN* tumours support this hypothesis. However, as our sample population did not include untreated patients, this hypothesis requires further evaluation.

The improved OS for patients with high *AURKA-CN* was most pronounced in our study among patients with *KRAS* wild-type tumours. Furthermore, the improvement in PFS on first-line and second-line chemotherapy was most pronounced in this specific patient population. The small sample size of this subset analysis, while demonstrating statistical significance, limits definitive conclusion and requires further validation in an independent data set. We did not observe any correlation between the presence of *KRAS* mutations and *AURKA-CN*, suggesting these may represent two independent pathways in the biology of CRC. By combining these two biomarkers, we may be able to identify a subgroup of patients with mCRC who exhibit increased response to therapy and superior outcomes. The improved response of patients with *KRAS* wild-type tumours treated with anti-EGFR therapy has been well documented in the literature (Cunningham *et al*, 2004; Karapetis *et al*, 2008; Van Cutsem *et al*, 2009; Douillard *et al*, 2010; Peeters *et al*, 2010; Bokemeyer *et al*, 2011). However in our study, among patients with *KRAS* wild-type tumours, the impact of high *AURKA-CN* level was most pronounced in patients who did *not* receive cetuximab. One unifying hypothesis may be that Aurora A overexpression confers sensitivity to chemotherapy and not to anti-EGFR therapy. Alternatively, the relatively small number of patients available for this subgroup analysis may preclude a meaningful assessment of this association. *In-vitro* work has demonstrated strong synergy between anti-EGFR drugs and novel agents targeting the Aurora pathway, which may potentially become a therapeutic approach (Astsaturov *et al*, 2010). Further research to evaluate the response to EGFR-targeted therapy in patients with high and low *AURKA-CN* may further define the interaction between these two pathways.

Many groups have studied AURKA protein expression by IHC in CRC and other cancers. In CRC, increased expression of AURKA protein by IHC has been noted in 19–30% of patients (Bischoff *et al*, 1998; Lam *et al*, 2008; Baba *et al*, 2009). In our study, IHC analysis was performed on approximately one third of the available samples, of which a third displayed positive staining (*n*=9; 33%). Our results demonstrated lack of staining of samples with low *AURKA-CN*, and positive staining in less than half of the samples with high *AURKA-CN*. Increased gene copy number is expected to result in increased activity of the product protein. Thus, the fact that most of the samples with high *AURKA-CN* were not found to have a positive stain suggests the possibility that despite high copy number AURKA may in fact be inactive in these cells due to post-transcriptional and post-translational regulation. Alternatively, this observation may raise questions regarding the sensitivity of IHC staining. IHC carries many challenges, most notably a variable pattern of staining and the presence of artifacts related to the fixation process. Differences in the preservation process between the samples may also result in various rates of protein degradation, which may affect the IHC protein expression. Furthermore, this assay is user dependent and may suffer from significant inter-reader variability. A PCR-based method is likely to be more accurate and sensitive in detecting increased activity of a pathway at the gene level.

Our study does have several limitations. As a retrospective review, we were limited by specimen availability, which may have introduced unexpected bias into the results. However, by using all specimens available during a specific timeframe, we attempted to minimise this impact. The small sample size limits our ability to draw clear conclusions regarding some of the analyses conducted in the study, such as the interaction between *AURKA-CN* level and the use of cetuximab. We also did not evaluate a validation cohort to support our chosen cutoff for the analysis of *AURKA-CN* level. However, all cutoffs selected demonstrated improved clinical outcome for patients with high compared with low *AURKA-CN*.

In summary, we demonstrated a high frequency of increased *AURKA-CN* in mCRC samples using a novel statistical methodology to evaluate the most appropriate cutoff for analysis. Moreover, our study is the first to demonstrate an association between high *AURKA-CN* and improved clinical outcome among patients with mCRC receiving chemotherapy, with a more pronounced association noted among patients with *KRAS* wild-type tumours. Additional study utilising tissue from larger randomised studies to distinguish the prognostic and predictive impact of *AURKA-CN* is warranted. Implications of these findings for future development of agents targeting Aurora kinase should be considered.

## Figures and Tables

**Table 1:** Patient and treatment characteristics

	**Full cohort (*N*=59)**	**Low *Aurora CN* ⩽2.6 (*N*=18)**	**High *Aurora CN* >2.6 (*N*=41)**	***P*-value**
Age, mean (s.d.)	58.6 (1.44)	58.6 (3.2)	58.7 (1.58)	0.97
				
*Gender, N (%)*				0.68
Male	37 (62.7)	12 (66.7)	25 (61.0)	
Female	22 (37.3)	6 (33.3)	16 (39.0)	
				
*Race, N (%)*				
Caucasian	51 (87.9)	14 (82.3)	37 (90.2)	0.41
AA	7 (12.1)	3 (17.7)	4 (9.8)	
				
*Stage at diagnosis, N (%)*				0.80
2	1 (1.9)	0 (0)	1 (2.7)	
3	10 (8.9)	3 (18.8)	7 (18.9)	
4	42 (79.3)	13 (81.2)	29 (78.4)	
				
**Clinical and treatment data for 53 patients *n*(%)**				
	**(*N*=53)**	**(*N*=16)**	**(*N*=37)**	
*No. of metastatic sites*				
1	30 (56.7)	11 (51.4)	19 (68.8)	0.28
2	20 (37.7)	16 (43.25)	4 (25.0)	
3	2 (3.8)	1 (2.7)	1 (6.25)	
4	1 (1.8)	1 (2.7)	0 (0.0)	
				
*First-line therapy (all patients received 5-FU or capecitabine with initial therapy)*				
Oxaliplatin, *N* (%)	45 (84.9)	11 (81.3)	34 (86.5)	0.63
Irinotecan, *N* (%)	5 (9.4)	2 (12.5)	3 (8.1)	0.63
Cetuximab, *N* (%)	19 (35.9)	7 (43.8)	12 (32.4)	0.54
Bevacizumab, *N* (%)	35 (66.0)	10 (62.5)	25 (67.6)	0.76
Bevacizumb+Cetuximab, *N* (%)	8 (15.0)	4 (25)	4 (10.8)	0.19
				
**Second-line therapy**				
	**(*N*=33)**	**(*N*=11)**	**(*N*=22)**	
5-FU or Capecitabine, *N* (%)	14 (42.4)	7 (63.6)	7 (31.8)	0.14
Irinotecan, *N* (%)	26 (78.7)	9 (81.8)	17 (77.3)	0.57
Oxaliplatin, *N* (%)	2 (6)	1 (9)	1 (4.5)	0.56
Bevacizumab, *N* (%)	11 (33.3)	8 (72.7)	3 (13.6)	0.001
Cetuximab/Panitumumab, *N* (%)	17 (51.5)	6 (54.5)	11 (50)	0.55
Third-line therapy	9 (27.2)	2 (18.2)	7 (31.8)	0.35

Abbreviations: AA=African American; CN=copy number.

**Table 2:** Survival analysis utilising various *AURKA-CN* cutoff values for the entire cohort

**Cutoff**	**Percentage of low *AURKA-CN***	**HR**	**s.e.**	***Z***	***P*>∣z∣**	**95% Confidence interval**	**Harrell's C index**	**Log-rank *P*-value**
2.5	24	0.377	0.193	−1.9	0.057	0.138–1.030	0.59	0.05
2.6	31	0.280	0.138	−2.57	0.01	0.106–0.738	0.63	0.0064
2.813	36	0.401	0.180	−2.03	0.042	0.166–0.969	0.64	0.04
3	41	0.461	0.205	−1.74	0.081	0.193–1.101	0.62	0.07
3.5	44	0.447	0.199	−1.8	0.071	0.187–1.072	0.62	0.06
4	52	0.507	0.229	−1.5	0.133	0.210–1.228	0.61	0.13
5	64	0.633	0.307	−0.94	0.347	0.245–1.640	0.58	0.34

Abbreviation: *AURKA-CN*=Aurora kinase A gene copy number.

**Table 3:** Progression-free survival on first-line chemotherapy by *KRAS* mutation status and receipt of cetuximab

	***N***	**Low *AURKA-CN* median PFS (months)**	**High *AURKA-CN* median PFS (months)**	**HR**	***P*-value**
*First-line chemotherapy*					
All patients	53	7.7 (95% CI: 3.3–14.5)	11.5 (95% CI: 8.9–20.6)	0.57 (95% CI: 0.29–1.1)	0.10
No cetuximab exposure	34	5.1 (95% CI: 1.4–14.5)	11.5 (95% CI: 8.9–20.7)	0.47 (95% CI: 0.20–1.11)	0.08
With cetuximab exposure	19	7.9 (95% CI: 0.7–XX^a^)	10.2 (95% CI: 4.5–XX^a^)	0.68 (95% CI: 0.21–2.17)	0.51
Odds ratio for cetuximab		0.70 (0.23–2.18) *P*=0.54	0.90 (0.39–2.06) *P*=0.80		
					
*First-line chemotherapy – KRAS wild type*					
All patients	38	5.13 (95% CI: 1.4–14.5)	17.6 (95% CI: 9.1–25.3)	0.43 (95% CI: 0.19–0.94)	0.04
No cetuximab exposure	24	4.4 (95% CI: 1.4–11.4)	14.0 (95% CI: 6.2–22.4)	0.24 (95% CI: 0.08–0.67)	0.01
With cetuximab exposure	14	9.6 (95% CI: 0.7–XX^a^)	17.6 (95% CI: 2.5–XX^a^)	0.71 (95% CI: 0.17–3.04)	0.65
Odds ratio for cetuximab		0.39 (0.10–1.55) *P*=0.18	0.78 (0.29–2.09) *P*=0.62		
					
*First-line chemotherapy – KRAS mutant*					
All patients	15	7.9 (95% CI: 7.7–XX^a^)	11.4 (95% CI: 4.2–34.2)	1.06 (95% CI: 0.29–3.93)	0.93
No cetuximab exposure	10	20.1 (95% CI: XX^a^–XX^a^)	11.4 (95% CI: 4.2–34.1)	2.5 (95% CI: 0.29–20.9)	0.46
With cetuximab exposure	5	7.7 (95% CI: 7.7–XX^a^)	5.3 (95% CI: 2.8–XX^a^)	1.1 (95% CI: 0.15–7.9)	0.95
Odds ratio for cetuximab		Number too small to estimate	1.36 (0.27–6.84) *P*=0.71		

Abbreviations: *AURKA-CN*=Aurora kinase A gene copy number; CI=confidence interval; HR=hazard ratio; PFS=progression-free survival.

a Not enough data to estimate the complete 95% CI.
